# Lysophosphatidic Acid Promotes Epithelial–Mesenchymal Transition in Kidney Epithelial Cells via the LPAR1/MAPK-AKT/KLF5 Signaling Pathway in Diabetic Nephropathy

**DOI:** 10.3390/ijms231810497

**Published:** 2022-09-10

**Authors:** Geon-Ho Lee, Jayeon Cheon, Donghee Kim, Hee-Sook Jun

**Affiliations:** 1 College of Pharmacy, Gachon Institute of Pharmaceutical Sciences, Gachon University, Incheon 21936, Korea; 2 Lee Gil Ya Cancer and Diabetes Institute, Gachon University, Incheon 21999, Korea; 3 Gachon Medical Research Institute, Gil Hospital, Incheon 21565, Korea

**Keywords:** diabetic nephropathy, lysophosphatidic acid receptor 1, epithelial–mesenchymal transition, Krüppel-like factor 5

## Abstract

The epithelial–mesenchymal transition (EMT) is a differentiation process associated with fibrogenesis in diabetic nephropathy (DN). Lysophosphatidic acid (LPA) is a small, naturally occurring glycerophospholipid implicated in the pathogenesis of DN. In this study, we investigated the role of LPA/LPAR1 signaling in the EMT of tubular cells as well as the underlying mechanisms. We observed a decrease in E-cadherin and an increase in vimentin expression levels in the kidney tubules of diabetic db/db mice, and treatment with ki16425 (LPAR1/3 inhibitor) inhibited the expression of these EMT markers. Ki16425 treatment also decreased the expression levels of the fibrotic factors fibronectin and alpha-smooth muscle actin (α-SMA) in db/db mice. Similarly, we found that LPA decreased E-cadherin expression and increased vimentin expression in HK-2 cells, which was reversed by treatment with ki16425 or AM095 (LPAR1 inhibitor). In addition, the expression levels of fibronectin and α-SMA were increased by LPA, and this effect was reversed by treatment with ki16425 and AM095 or by LPAR1 knockdown. Moreover, LPA induced the expression of the transcription factor, Krüppel-like factor 5 (KLF5), which was decreased by AM095 treatment or LPAR1 knockdown. The expression levels of EMT markers and fibrotic factors induced by LPA were decreased upon KLF5 knockdown in HK-2 cells. Inhibition of the extracellular signal-regulated kinase (ERK), c-Jun N-terminal kinase (JNK), and serine-threonine kinase (AKT) pathways decreased LPA-induced expression of KLF5 and EMT markers. In conclusion, these data suggest that LPA contributes to the pathogenesis of diabetic nephropathy by inducing EMT and renal tubular fibrosis via regulation of KLF5 through the LPAR1.

## 1. Introduction

Diabetic nephropathy (DN) is one of the major microvascular complications of diabetes and a major cause of end-stage renal disease [1,2]. Glomerular hypertrophy, matrix protein accumulation, and tubular injury are the major pathological features of DN [3,4]. Tubular interstitial fibrosis is a common final pathway leading to progressive renal injury in various conditions, including DN [5].

The epithelial–mesenchymal transition (EMT) is implicated in the major pathway leading to renal interstitial fibrosis in DN [6,7,8], and the EMT of renal tubular cells is the most common cause of renal dysfunction [9,10]. During EMT, epithelial cells lose several epithelial properties, such as E-cadherin, and acquire the characteristics typical of mesenchymal cells, such as vimentin [11]. 

Lysophosphatidic acid (LPA) is a small, naturally occurring glycerophospholipid that acts through G protein-coupled receptors (GPCRs; LPA receptors (LPARs) 1–6) [12]. LPA is associated with various processes, such as cell proliferation and migration, and the pathologies of several diseases, including neurological disorders, atherosclerosis, cardiovascular disease, fibrosis, obesity, and cancer [12,13,14]. Inhibition of LPA signaling attenuates fibrotic responses, such as fibroblast growth, collagen deposition, and activation of pro-fibrotic factors (transforming growth factor-β (TGF-β) and connective tissue growth factor), in skin, lung, and renal fibrosis [12,15,16]. A previous report confirmed that LPA levels were significantly increased in diabetic and high-fat-diet-induced obese mice [17,18]. The urinary levels of LPA and lysophosphatidylcholine, a precursor of LPA, are significantly increased in diabetic patients with DN than in those without DN [19]. In addition, we previously confirmed that LPA/LPAR1 signaling plays an important role in the development of DN and fibrosis in diabetic db/db and streptozotocin-induced diabetic mice [20,21]. However, the role of LPA/LPAR1 signaling in EMT in DN has not yet been studied.

Krüppel-like factor 5 (KLF5) is a basic transcription factor that binds to the GC box and regulates the transcription of multiple gene promoters and various cellular functions, such as the cell cycle, cell proliferation, apoptosis, migration, and differentiation [22]. In the kidney, KLF5 plays important roles in regulating mesangial cell proliferation, podocyte apoptosis, tubulointerstitial inflammation, and fibrosis [23]. Although KLF5 has been demonstrated to regulate tubular interstitial fibrosis in models of unilateral ureteral obstruction and subtotal nephrectomy (5/6 nephrectomy) [24,25], its role in tubular EMT in DN models is yet to be studied. 

In this study, we investigated the role of LPA in EMT in DN and the molecular mechanisms involved in LPA-induced EMT and fibrotic responses in renal tubular epithelial cells. We found that LPA induced EMT and renal fibrosis in tubular epithelial cells by regulating KLF5 via the mitogen-activated protein kinase (MAPK)–serine-threonine kinase (AKT) pathway through LPAR1, thus contributing to the pathogenesis of DN.

## 2. Results

### 2.1. Treatment with the LPAR1/3 Antagonist, ki16425, Inhibits the Change in the Expression Levels of EMT Markers and Fibrotic Factors in Renal Tubular Epithelial Cells of db/db Mice

**Figure 1:**
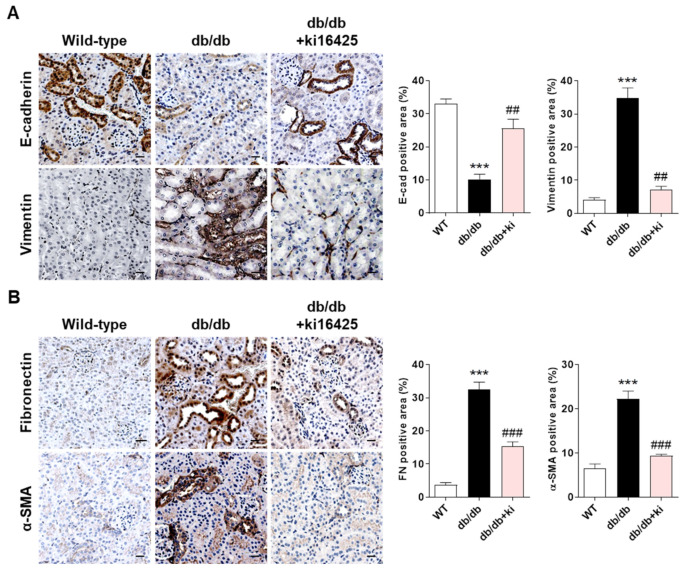
Treatment with the LPAR1/3 antagonist, ki16425, inhibits the change in the expression levels of EMT markers and fibrotic factors in renal tubular epithelial cells of db/db mice. The animal experiments are described in the Materials and Methods section. Immunohistochemical detection of (**A**) the EMT markers E-cadherin and vimentin, and (**B**) the fibrotic factors fibronectin (FN) and α-smooth muscle actin (α-SMA), in the kidney tissues was performed using specific antibodies and diaminobenzidine (DAB). Nuclei were counterstained with hematoxylin. The positive expression area of each target gene was quantified using ImageJ software. Representative images (left) and quantitative analysis bar graphs (right) are shown. Original magnification, 200×; scale bars, 20 μm; *n* = 3 mice per group. Data are presented as the mean ± standard error of the mean (SEM). *** *p* < 0.005 vs. wild-type (WT); ## *p* < 0.01, ### *p* < 0.005 vs. db/db.

The EMT was observed in the renal tubular epithelial cells of db/db mice during DN progression [10,26,27]. We previously reported that ki16425 administration in diabetic db/db mice significantly suppressed albuminuria and serum creatinine levels, and inhibited renal fibrosis without changes in blood glucose levels and body weight [20]. To determine whether ki16425 treatment affected EMT in renal tubular epithelial cells, we investigated the renal expression of EMT markers (E-cadherin and vimentin) in db/db mice. E-cadherin expression was significantly decreased in the renal tubules of db/db mice compared with those of wild-type mice; however, its expression was restored by treatment with ki16425 (Figure 1A). Conversely, vimentin expression was significantly increased in the tubules of db/db mice compared with those of wild-type mice, which was reversed by treatment with ki16425 (Figure 1A). Since the EMT of tubular epithelial cells is closely associated with the progression of renal fibrosis [28], we also investigated the renal expression of fibrotic factors, such as α-SMA and fibronectin, following ki16425 treatment. Similar to vimentin expression, α-SMA and fibronectin expression levels were significantly increased in the tubules of db/db mice compared with those in wild-type mice tubules; however, this effect was significantly suppressed by ki16425 treatment (Figure 1B). These results suggest that EMT in renal tubular epithelial cells is associated with the progression of LPA-mediated tubular fibrosis in DN.

### 2.2. LPAR1/3 or LPAR1 Inhibitor Treatment Inhibits LPA−Induced Changes in the Expression Levels of EMT Markers and Fibrotic Factors

**Figure 2:**
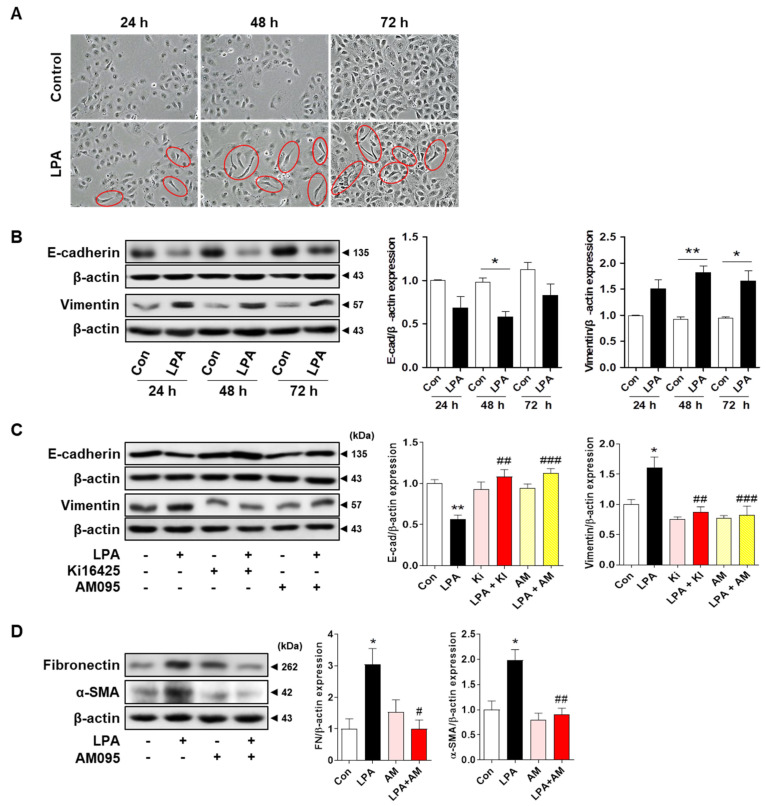
LPAR1/3 or LPAR1 inhibitor treatment inhibited LPA−induced changes in the expression levels of EMT markers and fibrotic factors. (**A**,**B**) HK-2 cells were treated with the vehicle or 20 μM LPA for 24, 48, and 72 h. (**A**) Cellular morphology was observed using light microscopy. Original magnification, 100×. Red circles indicate cells with an elongated spindle-shaped morphology. (**B**) Protein levels of E-cadherin and vimentin were measured using Western blotting, and representative images of the blots (left) and quantification bar graphs (right) are shown. (**C**,**D**) HK-2 cells were treated with 20 μM LPA in the presence or absence of 20 μM ki16425 (Ki) or 10 μM AM095 (AM) for 48 h. Protein levels of (**C**) E-cadherin (E-cad) and vimentin, and (**D**) fibronectin (FN) and α-smooth muscle actin (α-SMA) were measured using Western blotting, quantified using ImageJ software, and normalized to that of β-actin. Representative images (left) and quantitative analysis bar graphs (right) are shown (*n* = 3–4 independent experiments). Data are presented as the mean ± SEM. * *p* < 0.05, ** *p* < 0.01 vs. Con; # *p* < 0.05, ## *p* < 0.01, ### *p* < 0.005 vs. LPA.

The EMT induces changes in the cytoskeletal composition and cell polarity, resulting in spindle-shaped cell-forming arrangements [29]. To determine whether LPA induced EMT in renal tubular epithelial cells, we examined the morphological changes and EMT marker expression levels in LPA-treated HK-2 cells. We observed a morphological change from cobblestone-like to an elongated spindle shape in HK-2 cells from 24 to 72 h after LPA treatment (Figure 2A). We examined the expression levels of E-cadherin and vimentin to confirm whether the changes in cell shape were associated with changes in the expression levels of EMT markers. After 48 h of LPA treatment, the expression of E-cadherin was significantly decreased, while that of vimentin was significantly increased. This phenomenon was continuously observed for 72 h after treatment with LPA (Figure 2B).

To determine whether LPAR1 was involved in LPA-induced EMT in tubular epithelial cells, we measured the expression levels of E-cadherin and vimentin in HK-2 cells after co-treatment with LPA and the LPAR1/3 antagonist, ki16425, or a specific LPAR1 inhibitor, AM095. On the basis of the preliminary experimental results in HK-2 cells, we used 20 μM ki16425 to suppress the expression of LPA-induced EMT markers (data not shown). Treatment with ki16425 or AM095 significantly inhibited the LPA-induced decrease in E-cadherin expression (Figure 2C). Conversely, LPA significantly increased vimentin expression, and treatment with ki16425 or AM095 attenuated this increase (Figure 2C). In addition, the expression levels of fibrotic factors (α-SMA and fibronectin) were significantly increased by the LPA treatment, which was inhibited by AM095 treatment (Figure 2D). These results suggest that LPA induces EMT mainly via LPAR1 due to the similar effects of LPA-induced EMT inhibition by ki16425 and AM095.

### 2.3. LPA Induces the Expression of EMT Markers and Fibrotic Factors through LPAR1 in HK-2 Cells

**Figure 3:**
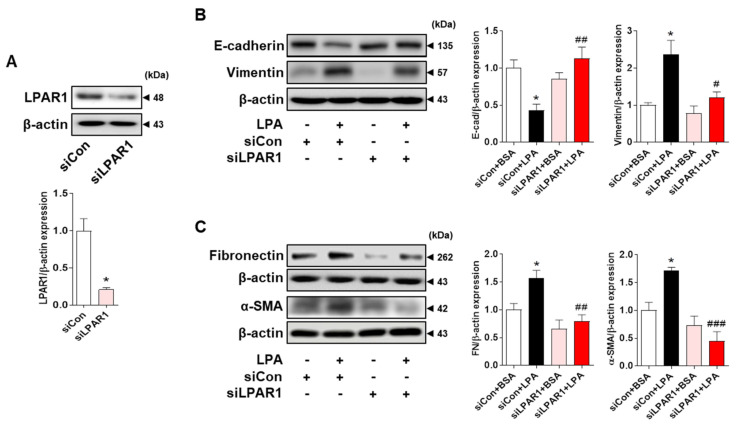
LPAR1 knockdown inhibited LPA−induced changes in the expression levels of EMT markers and fibrotic factors in HK-2 cells. HK-2 cells were transfected with control siRNA (siCon) or LPAR1 siRNA (siLPAR1) for 6 h. Next, the medium was replaced with a serum-free medium (SFM), then the cells were incubated for 16–18 h and treated with 20 μM LPA for 48 h. Protein levels of (**A**) LPAR1, (**B**) E-cadherin and vimentin, and (**C**) fibronectin and α-SMA were analyzed via Western blotting, quantified using ImageJ software, and normalized to that of β-actin. Representative images (left) and quantitative analysis bar graphs (right) are shown (*n* = 3 independent experiments). Data are presented as the mean ± SEM. * *p* < 0.05 vs. siCon or siCon + BSA; # *p* < 0.05, ## *p* < 0.01, ### *p* < 0.005 vs. siCon + LPA.

To confirm that LPAR1 mediated the LPA-induced expression of EMT markers and fibrotic factors, we transfected HK-2 cells with LPAR1 siRNA and examined the expression levels of EMT markers and fibrotic factors after LPA treatment. LPAR1 expression was significantly lower in LPAR1 siRNA-transfected cells than in control siRNA-transfected cells (Figure 3A). LPAR1 knockdown significantly increased E-cadherin expression and significantly decreased vimentin expression compared with that in LPA-treated control siRNA-transfected cells (Figure 3B). In addition, the LPA-induced expression of fibrotic factors (α-SMA and fibronectin) was significantly decreased by LPAR1 knockdown (Figure 3C). These results indicate that LPA/LPAR1 signaling is involved in the EMT and the fibrotic response in HK-2 cells.

### 2.4. LPA/LPAR1 Signaling Induces KLF5 Expression

**Figure 4:**
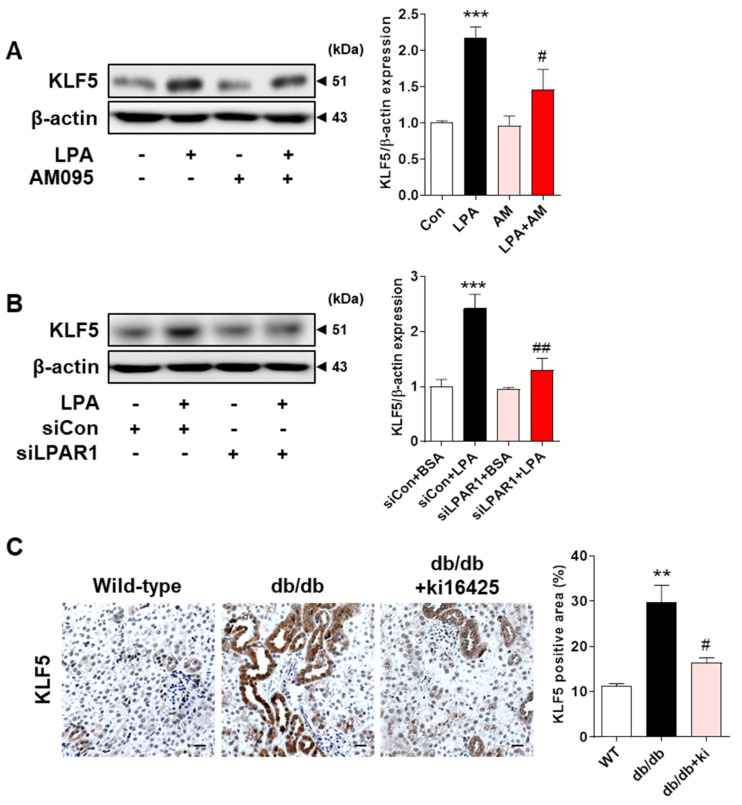
LPA induced KLF5 expression via LPAR1 in HK-2 cells and db/db mice. (**A**) HK-2 cells were treated with 20 μM LPA in the presence or absence of 10 μM AM095 for 30 min. (**B**) HK-2 cells were transfected with a control siRNA (siCon) or LPAR1 siRNA (siLPAR1) and treated with 20 μM LPA for 30 min. (**A**,**B**) Protein levels of KLF5 were analyzed via Western blotting, quantified using ImageJ software, and normalized to that of β-actin. Representative images (left) and quantitative analysis bar graphs (right) are shown (*n* = 3 independent experiments). Data are presented as the mean ± SEM. *** *p* < 0.005 vs. Con or siCon+BSA; # *p* < 0.05, ## *p* < 0.01 vs. LPA or siCon+LPA. (**C**) The animal experiments are described in the Materials and Methods section. Immunohistochemical detection of KLF5 was performed in the kidney tissues using DAB. The nuclei were counterstained with hematoxylin. The positive expression area of KLF5 was quantified using ImageJ software. Representative images (left) and quantitative analysis bar graph (right) are shown. Original magnification, 200×; scale bars, 20 μm; *n* = 3 mice per group. Data are presented as the mean ± SEM. ** *p* < 0.01 vs. wild-type (WT); # *p* < 0.05 vs. db/db.

KLF5 is a zinc finger-containing transcription factor involved in important biological processes, including cell transformation, proliferation, and carcinogenesis [30,31]. Since KLF5 contributes to the induction of EMT [32], we examined whether LPA treatment could induce its expression in HK-2 cells. We found that LPA significantly increased KLF5 expression, which was inhibited by AM095, an LPAR1 inhibitor (Figure 4A). Knockdown of LPAR1 by LPAR1 siRNA also suppressed the LPA-induced increase in KLF5 expression (Figure 4B), indicating that KLF5 expression was induced by LPA via LPAR1. In addition, we confirmed that the expression levels of KLF5 were significantly increased in the tubules of diabetic db/db mice compared with those in wild-type mice tubules, and this increase was significantly inhibited by the ki16425 treatment (Figure 4C). These results indicate that the LPA/LPAR1 signaling pathway regulates the expression of KLF5, which may contribute to EMT in the kidney tubular epithelial cells of db/db mice.

### 2.5. LPA−Induced Expression of EMT Markers and Fibrotic Factors Is Mediated by KLF5 in HK-2 Cells

**Figure 5:**
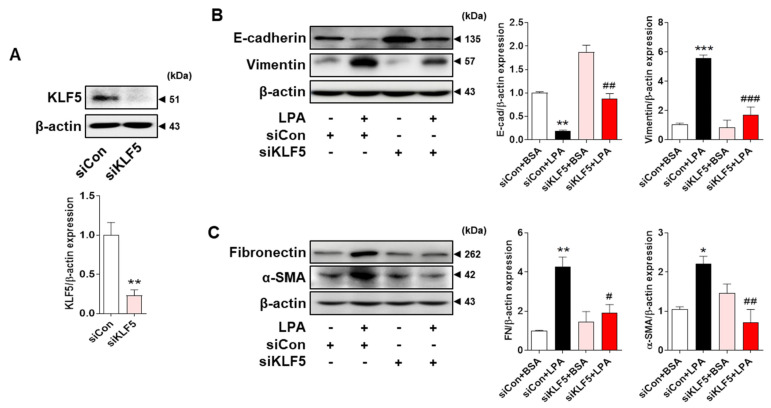
KLF5 mediates LPA−induced expression of EMT markers and fibrotic factors in HK-2 cells. HK-2 cells were transfected with a control siRNA (siCon) or LPAR1 siRNA (siLPAR1) for 6 h. Next, the medium was replaced with SFM for 16–18 h, and the cells were treated with 20 μM LPA for 48 h. Protein levels of (**A**) KLF5, (**B**) E-cadherin and vimentin, and (**C**) fibronectin and α-SMA were analyzed via Western blotting, quantified using ImageJ software, and normalized to that of β-actin. Representative images (left) and quantitative analysis bar graphs (right) are shown (*n* = 3 independent experiments). Data are presented as the mean ± SEM. * *p* < 0.05, ** *p* < 0.01, *** *p* < 0.005 vs. siCon or siCon + BSA; # *p* < 0.05, ## *p* < 0.01, ### *p* < 0.005 vs. siCon + LPA.

To determine whether LPA-induced KLF5 regulated the EMT in tubular epithelial cells, we investigated LPA-induced EMT after KLF5 knockdown using siRNA in HK-2 cells. We found that KLF5 expression was significantly decreased by KLF5 siRNA transfection in HK-2 cells (Figure 5A). Moreover, the expression of E-cadherin decreased while that of vimentin increased after LPA treatment in siCon-transfected cells; however, this change in expression was inhibited in siKLF5-transfected HK-2 cells (Figure 5B). LPA-induced expression of the fibrotic factors fibronectin and α-SMA was also inhibited in the siKLF5 group (Figure 5C). These results suggest that KLF5 plays important roles in the regulation of LPA-induced expression of EMT markers and fibrotic factors.

### 2.6. LPA−Induced KLF5 Expression Is Regulated by MAPK and AKT Signaling Pathways in HK-2 Cells

**Figure 6:**
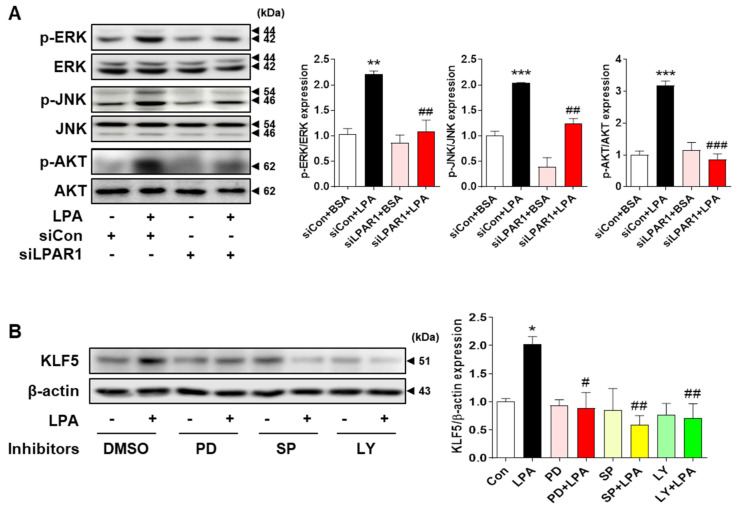
LPA−induced KLF5 expression is regulated by the MAPK and AKT signaling pathways in HK-2 cells. (**A**) HK-2 cells were transfected with a control siRNA (siCon) or LPAR1 siRNA (siLPAR1) and treated with or without 20 μM LPA for 30 min. (**B**) HK-2 cells were pretreated with the vehicle (dimethyl sulfoxide (DMSO)), PD 98059 (PD; an extracellular signal-regulated kinase (ERK) inhibitor; 10 μM), SP600125 (SP; a c-Jun N-terminal kinase (JNK) inhibitor; 10 μM), or LY 294002 (LY; a phosphoinositide 3-kinase (PI3K)/serine-threonine kinase (AKT) inhibitor; 2.5 μM) for 1 h, and subsequently treated with or without 20 μM LPA for 30 min. Protein levels of (**A**) p-ERK, p-JNK, and p-AKT, and (**B**) KLF5 were analyzed via Western blotting, quantified using ImageJ software, and normalized to those of total ERK, total JNK, total AKT, and β-actin, respectively. Representative images (left) and quantitative analysis bar graphs (right) are shown (*n* = 3 independent experiments). Data are presented as the mean ± SEM. * *p* < 0.05, ** *p* < 0.01, *** *p* < 0.005 vs. siCon + BSA or Con; # *p* < 0.05, ## *p* < 0.01, ### *p* < 0.005 vs. siCon + LPA or LPA.

LPA induces the phosphorylation of ERK, JNK, and AKT in tubular cells in injured kidneys with increased fibrosis [33,34]. Therefore, we examined whether these signaling pathways were involved in KLF5 expression in this study. We observed that ERK, JNK, and AKT were activated by LPA treatment in HK-2 cells. However, knockdown of LPAR1 decreased the expression levels of phosphorylated ERK, JNK, and AKT in cells compared with those in siCon-transfected HK-2 cells (Figure 6A), suggesting that the phosphorylation of ERK, JNK, and AKT is regulated via the LPA/LPAR1 pathway. We then investigated whether LPA-induced KLF5 expression was regulated by the ERK, JNK, and AKT pathways. HK-2 cells were pretreated with the inhibitors of each signaling molecule, and KLF5 expression was determined after LPA treatment. LPA-induced KLF5 expression was significantly decreased by treatment with the ERK (PD 98059), JNK (SP600125), and PI3K/AKT (LY 294002) inhibitors (Figure 6B). These results suggest that LPA-induced KLF5 expression is regulated by the ERK, JNK, and AKT signaling pathways.

### 2.7. LPA−Induced EMT Marker Expression Is Mediated via the MAPK and AKT Signaling Pathways

**Figure 7:**
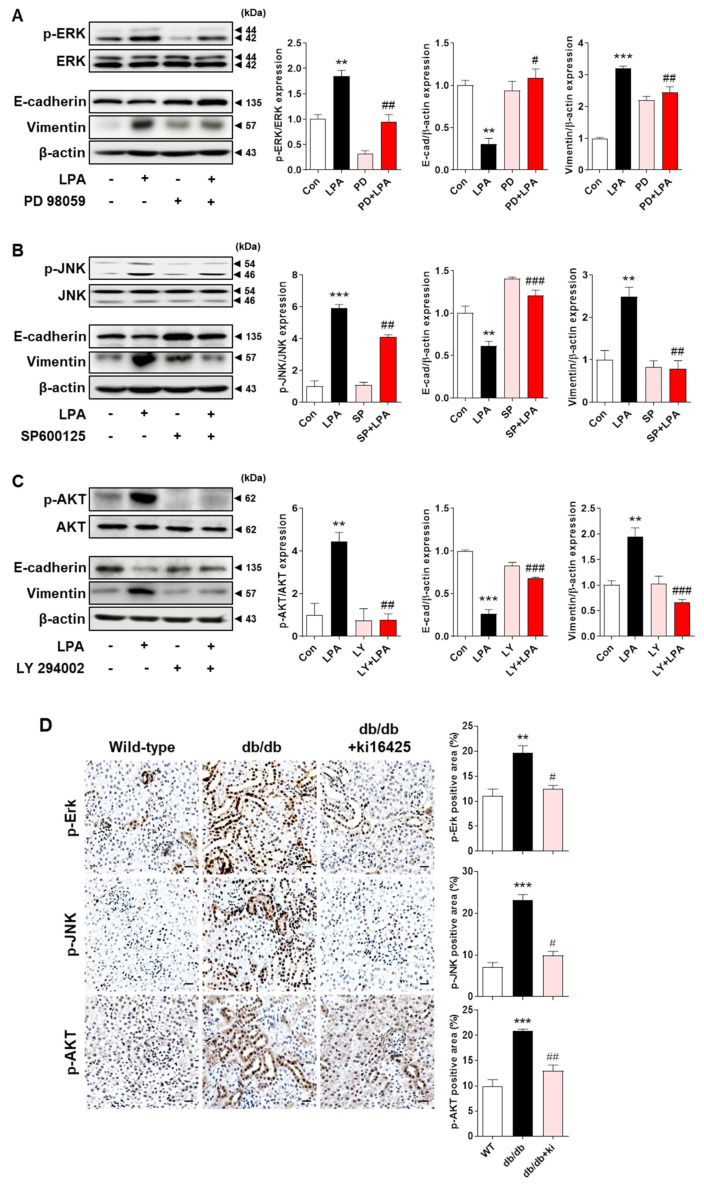
LPA induced EMT marker expression via the MAPK and AKT signaling pathways. (**A**–**C**) HK-2 cells were pretreated with the vehicle (DMSO), 10 µM PD 98059 (PD; an ERK inhibitor) (**A**), 10 µM SP600125 (SP; a JNK inhibitor) (**B**), or 2.5 µM LY 294002 (LY; a PI3K/AKT inhibitor) (**C**) for 1 h and subsequently treated with 20 µM LPA for 30 min (for p-ERK, p-JNK, and p-AKT) or 48 h (for E-cadherin and vimentin). Protein levels of E-cadherin, vimentin, p-ERK, p-JNK, and p-AKT were analyzed via Western blotting, quantified using ImageJ software, and normalized to those of β-actin, total ERK, total JNK, and total AKT, respectively. Representative images (left) and graph analysis bar graphs (right) are shown (*n* = 3 independent experiments). Data are presented as the mean ± SEM. ** *p* < 0.01, *** *p* < 0.005 vs. Con; # *p* < 0.05, ## *p* < 0.01, ### *p* < 0.005 vs. LPA. (**D**) The animal experiments are described in the Materials and Methods section. Immunohistochemical detection of p-ERK, p-JNK, and p-AKT was performed in the kidney tissues using specific antibodies and DAB. The nuclei were counterstained with hematoxylin. The positive expression area of each target gene was quantified using ImageJ software. Representative images (left) and quantitative analysis bar graph (right) are shown. Original magnification, 200×; scale bars, 20 μm; *n* = 3 mice per group. Data are presented as the mean ± SEM. ** *p* < 0.01, *** *p* < 0.005 vs. wild-type (WT); # *p* < 0.05, ## *p* < 0.01 vs. db/db.

Since we found that LPA-induced KLF5 expression was regulated by the ERK, JNK, and AKT signaling pathways, we confirmed whether these signaling pathways subsequently affected the expression of EMT markers in LPA-treated HK-2 cells. Treatment with LPA significantly reduced the expression of E-cadherin and increased the expression of vimentin, which was reversed by treatment with the ERK, JNK, and PI3K/AKT inhibitors (Figure 7A–C). Expression levels of p-ERK, p-JNK, and p-AKT were also significantly increased in the tubules of db/db mice compared with those in wild-type mice, and were significantly inhibited by the ki16425 treatment (Figure 7D). Taken together, these results suggest that the ERK, JNK, and AKT signaling pathways are involved in LPA-induced EMT by regulating KLF5 expression.

## 3. Discussion

DN is a common complication of diabetes [1,2]. DN is characterized by the accumulation of extracellular matrix proteins, leading to glomerulosclerosis and tubular interstitial fibrosis; however, the underlying pathophysiological mechanisms remain unclear [35,36]. EMT is one of the main mechanisms, and early changes in tubular epithelial cells are important factors in DN progression [6,8,37].

The importance of plasma LPA in patients with renal failure was first proposed in the late 1990s [38], and an increase in LPA levels was detected in the urine of patients with DN [19]. In addition, serum autotaxin levels are associated with proteinuria and renal lesions in patients with Type 2 diabetes and biopsy-proven DN [39]. A recent study showed that the treatment of endothelial nitric oxide synthase-knockout db/db mice with BMS002, an LPAR1/3 antagonist, attenuated the development of DN by providing protection against podocyte loss [40]. In addition, our previous study showed that autotaxin and LPAR1 expression levels were significantly increased in the renal cortex of db/db mice, and inhibition of LPAR1/3 by treatment with ki16425 improved renal fibrosis [20]. An LPAR1 inhibitor, AM095, also attenuates DN in streptozotocin-induced diabetic mice by inhibiting toll-like receptor 4/nuclear factor-κB signaling and downregulating NADPH oxidase expression [21]. Although we previously studied the role of LPA and the associated molecular mechanisms, mostly in mesangial cells [20,21,41,42], its role in tubular epithelial cells remained unclear. Therefore, in this study, we investigated the role of LPA and the molecular mechanisms underlying LPA-induced EMT in renal proximal tubular epithelial cells.

During the EMT, epithelial cells lose intercellular apical–basal polarity and undergo changes in their signaling mechanisms, resulting in reorganization of the cytoskeleton and reprogramming of gene expression [43,44]. Characteristics of EMT include a decrease in E-cadherin, tight junction proteins, and cytokeratin levels and increases in mesenchymal marker levels, such as vimentin and N-cadherin [29]. Our study showed that the decrease in E-cadherin and the increase in vimentin expression in the kidneys of db/db mice were inhibited by ki16425 treatment (Figure 1A). In addition, the expression levels of the fibrotic factors α-SMA and fibronectin were increased in the kidney tubular epithelial cells of db/db mice and decreased after treatment with ki16425 (Figure 1B), suggesting that LPA-mediated EMT may be involved in fibrosis in DN. 

We observed a change in the cell shape from a cobblestone shape to a spindle shape after LPA treatment in HK-2 cells (Figure 2A). In addition, LPA treatment decreased E-cadherin expression and increased vimentin expression (Figure 2B). Our experimental results are consistent with the reported findings that LPA reduces E-cadherin expression in ovarian cancer cells [45]. Consistent with the in vivo experiments, the expression of LPA-induced EMT markers in HK-2 cells was suppressed by treatment with ki16425. Ki16425 inhibits LPAR1 and LPAR3 [46]. The expression of LPAR1, but not LPAR3, was detected in tubular epithelial cells in mice [47]. Therefore, we examined the expression of EMT markers after treatment with AM095, a specific LPAR1 inhibitor, and found that the degree of inhibition was similar to that of ki16425. Consistent with this result, AM095 treatment suppressed the LPA-induced expression of α-SMA and fibronectin (Figure 2D). To confirm whether these effects were truly mediated through LPAR1, we used an LPAR1 knockdown system. The expression levels of LPA-induced EMT and fibrotic factors were also suppressed by LPAR1 knockdown, indicating that the LPA-induced EMT and fibrotic responses were induced by LPAR1 in HK-2 cells (Figure 3B,C).

KLF5 is a transcription factor that contains zinc fingers and is highly expressed in proliferating epithelial cells and adult tissues [30,31]. A previous report showed that KLF5 expression was increased in renal tubular cells in an in vivo model of unilateral ureteral obstruction and promoted renal fibrosis [48]. KLF5 induces TGF-β1 upregulation, contributing to the induction of EMT [32] and tubulointerstitial fibrosis [25]. Therefore, we hypothesized that KLF5 is involved in LPA-induced EMT in HK-2 cells. In our experiments, AM095 treatment or LPAR1 knockdown suppressed the expression of KLF5, indicating that LPA-induced expression of KLF5 is mediated by LPA/LPAR1 signaling (Figure 4A,B). We also observed that the expression of KLF5 was increased in the kidney tubular epithelial cells of db/db mice and decreased in ki16425-treated db/db mice (Figure 4C). LPA increases the expression of KLF5 to increase cell proliferation in colon cancer cells, and this LPA-induced KLF5 expression is mediated by LPAR2 and LPAR3 [49]. These results indicate that different LPA receptors may be involved in LPA-induced KLF5 expression, depending on the cell type.

KLF5 knockdown experiments clearly showed that the LPA-induced expression of EMT markers and fibrotic factors is regulated by KLF5 in HK-2 cells (Figure 5B,C). Our results were consistent with those of a previous study showing that KLF5 regulates E-cadherin expression by binding directly to the slug promoter in immortalized mammary epithelial cells [50] and enhances vimentin expression via activation of the nuclear factor-kB pathway in thyroid cancer cells [51,52]. Taken together, these results suggest that LPA/LPAR1 signaling induces KLF5 expression and subsequently regulates the expression of the EMT and fibrotic factors. 

LPA induces various signaling pathways, including the ERK, JNK, and PI3K/AKT [33,34] pathways, during EMT induction in kidney tubular epithelial cells [53,54,55,56]. ERK activation is involved in high-glucose-induced EMT in HK-2 cells [53]. In HK-2 cells and a rat unilateral ureteral obstruction model of renal fibrosis, pentraxin 3 upregulated EMT by activating JNK signaling [54]. High-glucose-induced reactive oxygen species production induces EMT via the PI3K/AKT pathway in NRK-52E cells and in STZ-induced DN [55]. Therefore, we investigated whether these signaling molecules were activated by LPA in HK-2 cells. The phosphorylation of ERK, JNK, and AKT was increased by LPA treatment, which was inhibited by LPAR1 knockdown (Figure 6A). As we found that the expression levels of EMT and fibrotic factors were regulated by KLF5, we examined whether KLF5 expression was regulated by signaling pathways in HK-2 cells. Our results revealed that the LPA-induced expression of KLF5 and EMT markers was inhibited by treatment with each signaling inhibitor (Figure 6 and Figure 7), indicating that LPA-induced KLF5 expression is mediated by the ERK, JNK, and AKT signaling pathways via LPAR1. Consistently, the increased expression levels of p-ERK, p-JNK, and p-AKT in tubular epithelial cells of the kidneys of db/db mice were reduced by ki16425 treatment (Figure 7D). These results indicate that the ERK, JNK, and AKT signaling pathways are activated by LPA/LPAR1, contributing to the EMT response.

Although we found that LPAR1-mediated signaling plays a major role in LPA-induced EMT in tubular epithelial cells (HK-2 cells), the lack of in vivo studies using LPAR1-specific inhibitors is a limitation of this study. Further investigation of the effects of LPAR1-specific inhibitors on EMT in tubular epithelial cells will help to confirm the role of LPAR1 in tubular epithelial cells in the pathogenesis of DN. In addition, validation of LPAR1 expression in renal tubular epithelial cells in patients with DN will add significance for clinical applications.

**Figure 8:**
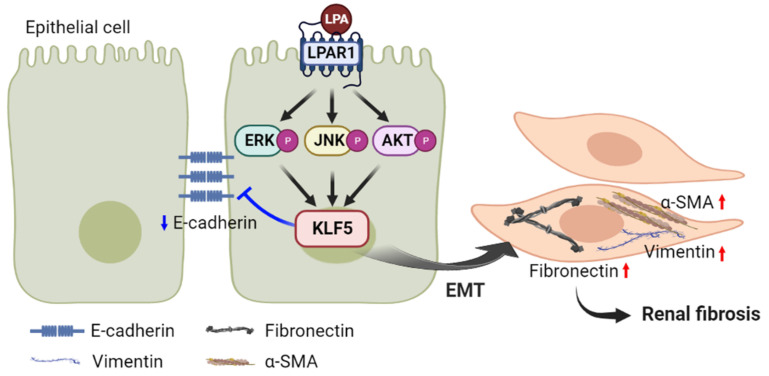
Schematic diagram of the mechanism by which LPA induces KLF5-mediated EMT and fibrotic responses via LPAR1/MAPK-AKT signaling in renal tubular epithelial cells. Created with BioRender.com. Red and blue arrows indicate upregulated and downregulated responses, respectively.

In summary, our results showed that LPA induced the activation of ERK, JNK, and PI3K/AKT signaling via LPAR1; increased the expression of KLF5; and subsequently induced the expression of EMT and renal fibrotic factors (Figure 8). This pathway may result in EMT and renal fibrosis in tubular epithelial cells, thereby contributing to the pathogenesis of DN.

## 4. Materials and Methods

### 4.1. Experimental Animals

Male diabetic db/db (BKS.Cg-leprdb/leprdb; C57BLKS/J) mice were obtained from Jackson Laboratories (Bar Harbor, ME, USA) at 7 weeks of age. Age-matched nondiabetic wild-type (BKS.Cg-lepr+/lepr+) mice were used as controls. One week after adaptation, the mice were intraperitoneally injected with ki16425 (10 mg/kg daily; #orb181043; Biorbyt Ltd., Cambridge, UK) or the vehicle for 8 weeks. The mice were sacrificed, and kidney tissues were obtained. All animal experiments were approved by the Institutional Animal Care and Use Committee of the Lee Gil Ya Cancer and Diabetes Institute, Gachon University (Incheon, Korea; LCDI-2016-0080, LCDI-2018-0115) [20].

### 4.2. Immunohistochemical Staining

Kidney tissues were fixed with 10% neutral buffered formalin (Sigma-Aldrich, St. Louis, MO, USA) after removal of the renal capsule, embedded in paraffin, and sliced into sections 4 μm thick. Sections were deparaffinized with xylene and rehydrated in a series of graded ethanol solutions. Heat-induced antigen retrieval and permeabilization were performed as previously described [42]. The sections were incubated with primary antibodies diluted 1:200 in an antibody diluent (Dako North America, Inc., Carpinteria, CA, USA). The following primary antibodies were used: E-cadherin (#14472; Cell Signaling Technology (CST), Boston, MA, USA), vimentin (#5741; CST), fibronectin (NBP1-91258; Novus Biologicals, Littleton, CO, USA), α-smooth muscle actin (α-SMA; #19245; CST), Krüppel-like factor 5 (KLF5; SAB2701988; Sigma-Aldrich), phospho-p44/42 MAPK (extracellular signal-regulated kinase (ERK)-1/2) (Thr202/Tyr204) (#9101; CST), phospho-SAPK/c-Jun N-terminal kinase (JNK) (Thr183/Tyr185) (#9251; CST), and p-AKT1/2/3 (C-11) (sc-514032; Santa Cruz Biotechnology, Santa Cruz, CA, USA). Immunohistochemical staining was performed using Polink-2 Plus horseradish peroxidase (HRP) rabbit with a DAB kit (D39-18; GBI Labs. Inc., Bothell, DC, USA) to detect vimentin, α-SMA, fibronectin, KLF5, p-ERK, and p-JNK levels, or using Polink-2 Plus HRP Mouse with a DAB Kit (D37-18, GBI Labs. Inc.) to detect E-cadherin and p-AKT levels, according to the manufacturer’s protocol. Nuclei were counterstained with hematoxylin (Sigma-Aldrich). The sections were subsequently mounted and observed under a microscope (AXIO Imager Z1, Carl Zeiss Inc., Oberkochen, Germany) at the Core-facility for Cell to In-vivo imaging. The positive expression area of each target gene was quantified using ImageJ software (Version 1.51; National Institutes of Health, Bethesda, MD, USA). Quantification was performed after converting the DAB staining images to 8-bit grayscale, and the percentage of expression reflected the percentage of the positively stained area in the image [57].

### 4.3. Cell Culture and Treatment

The human renal proximal tubular cell line, Human Kidney 2 (HK-2), was purchased from the American Type Culture Collection (CRL-2190; Rockville, MD, USA). HK-2 cells were maintained in the Roswell Park Memorial Institute-1640 medium (Welgene, Daegu, Korea) containing 5% fetal bovine serum (Gibco, Grand Island, NY, USA) and 1% penicillin–streptomycin (Welgene) at 37 °C in a humidified atmosphere of 5% CO_2_ and 95% air.

To investigate the effect of LPA, HK-2 cells (2.5 × 10^5^ cells in a 90 mm dish or 1 × 10^5^ cells in a 60 mm dish) were seeded and cultured for 24 h. According to our previous report [20], the medium was replaced with a serum-free medium (SFM) containing 0.1% fatty-acid-free bovine serum albumin (BSA; Sigma-Aldrich) and incubated for 17–18 h. The cells were then treated with BSA-conjugated-LPA (20 µM; 18:1 Lyso PA (1-oleoyl-2-hydroxy-sn-glycero-3-phosphate); Avanti Polar Lipids, Birmingham, AL, USA) for 24–72 h to examine the morphological changes and EMT marker expression levels. We selected 48 h for the subsequent studies, as the LPA-induced changes in the expression of EMT markers were significant at this period. To determine whether LPAR1 was involved, HK-2 cells were treated with 20 µM LPA in the presence or absence of 20 µM ki16425 or 10 µM AM095 for 48 h. Equal amounts of BSA solution for LPA and dimethyl sulfoxide (DMSO) for ki16425 and AM095 were used as the vehicles. To determine the signaling mechanisms involved, HK-2 cells were pretreated with the vehicle (DMSO), 10 µM PD 98059 (an ERK inhibitor; Sigma-Aldrich), 10 µM SP600125 (a JNK inhibitor; Sigma-Aldrich), or 2.5 µM LY 294002 (a phosphoinositide 3-kinase (PI3K)/AKT inhibitor; Sigma-Aldrich) for 1 h, followed by treatment with 20 µM LPA for 30 min or 48 h.

### 4.4. Transfection

HK-2 cells (2.5 × 10⁵ cells/90 mm dish) were seeded and transiently transfected with LPAR1 small interfering RNA (siRNA) (Bioneer Inc., Daejeon, Korea), KLF5 siRNA (Bioneer Inc.), or scrambled siRNA (Bioneer Inc.) using Lipofectamine RNAiMAX reagent (Invitrogen, Carlsbad, CA, USA), according to the manufacturer’s instructions. After 6 h of transfection, the medium was replaced with SFM and incubated for 17–18 h, and the cells were treated with the vehicle or 20 μM LPA for 30 min or 48 h.

### 4.5. Western Blotting

Total cellular protein extraction and Western blotting were performed as previously described [58]. For each target protein, an equal amount of the lysate sample (10–50 µg total) and the following primary antibodies were used: E-cadherin (1:1000, #14472; CST), vimentin (1:1000, #5741; CST), fibronectin (1:1000, NBP1-91258; Novus Biologicals), α-SMA (1:1000, #19245; CST), EDG2/LPA-1 (1:1000, ab23698; Abcam, Cambridge, UK), KLF5 (1:1000, SAB2701988; Sigma-Aldrich), phospho-p44/42 MAPK (ERK1/2) (Thr202/Tyr204) (1:2000, #9101; CST), p44/42 MAPK (ERK1/2) (1:2000, #9102; CST), phospho-SAPK/JNK (Thr183/Tyr185) (1:1000, #9251; CST), SAPK/JNK (1:1000, #9252; CST), p-AKT1/2/3 (C-11) (1:1000, sc-514032; Santa Cruz Biotechnology), AKT1/2/3 (5C10) (1:1000, sc-81434; Santa Cruz Biotechnology), and β-actin (1:3000, sc-47778; Santa Cruz Biotechnology). The membranes were then incubated with HRP-conjugated secondary goat anti-mouse (Invitrogen) or goat anti-rabbit (Jackson ImmunoResearch, West Grove, PA, USA) antibodies for 1 h at 25 °C. β-actin was used as the loading control. Three or four separate experiments were performed using different samples. The blots were developed using the Immobilon-Western chemiluminescent HRP substrate (Millipore Corp., Billerica, MA, USA) and visualized using the LAS4000 imaging system (Fujifilm Corp., Tokyo, Japan) or Amersham ImageQuant 800 systems (Cytiva Life Sciences (formerly GE Healthcare Life Sciences), MA, USA). Protein bands were quantified using ImageJ software (National Institutes of Health).

### 4.6. Statistical Analysis

Data are presented as the mean ± standard error of the mean (SEM). Statistical analyses were performed using Student’s *t*-test for comparisons between two groups and one-way analysis of variance (ANOVA) with Tukey’s multiple comparison for comparisons among multiple groups using GraphPad Prism version 7.03 (GraphPad Software Inc., San Diego, CA, USA). Statistical significance was set at *p* < 0.05.

## Data Availability

The data used to support the findings of this study are available from the corresponding author upon request.
